# Top 10 public health challenges to track in 2023: Shifting focus beyond a global pandemic

**DOI:** 10.1002/puh2.86

**Published:** 2023-05-02

**Authors:** Don Eliseo Lucero‐Prisno, Deborah Oluwaseun Shomuyiwa, M. B. N. Kouwenhoven, Thinley Dorji, Goodness Ogeyi Odey, Adriana Viola Miranda, Isaac Olushola Ogunkola, Yusuff Adebayo Adebisi, Junjie Huang, Lin Xu, Joseph Christian Obnial, Aminath Huda, Sarawut Thepanondh, Manuel Millar Dayrit, Salvador B. Evardone, M. D. Lamawansa, Samrawit Solomon Ethiopia, Lydia Aziato, Philip Baba Adongo, Mohamed Hindolo Samai, Fernando B. Garcia, Joselito F. Villaruz, Indika Mahesh Karunathilake, Hao Li, Patrick Alain Azanza, Ian Findlay, Martin C. S. Wong

**Affiliations:** ^1^ Department of Global Health and Development London School of Hygiene and Tropical Medicine London UK; ^2^ Faculty of Management and Development Studies University of the Philippines Open University Los Baños Laguna Philippines; ^3^ Faculty of Public Health Mahidol University Bangkok Thailand; ^4^ Faculty of Pharmacy University of Lagos Lagos Nigeria; ^5^ Global Health Focus Africa Lagos Nigeria; ^6^ Department of Physics Xi'an Jiaotong‐Liverpool University Suzhou China; ^7^ Department of Internal Medicine Central Regional Referral Hospital Gelephu Bhutan; ^8^ Department of Public Health University of Calabar Calabar Nigeria; ^9^ Global Health Focus Asia Bandung Indonesia; ^10^ Nuffield Department of Population Health University of Oxford Oxford UK; ^11^ JC School of Public Health and Primary Care Faculty of Medicine Chinese University of Hong Kong Hong Kong SAR China; ^12^ Department of Thoracic Surgery The First Affiliated Hospital School of Medicine Zhejiang University Hangzhou Zhejiang China; ^13^ East Avenue Medical Center Quezon Philippines; ^14^ School of Medicine The Maldives National University Malé Maldives; ^15^ Ateneo School of Medicine and Public Health Manila Philippines; ^16^ Eastern Visayas Regional Medical Center Tacloban City Philippines; ^17^ Department of Surgery Teaching Hospital Peradeniya Faculty of Medicine University of Peradeniya Kandy Sri Lanka; ^18^ School of Public Health St. Paul's Hospital Millennium Medical College Addis Ababa Ethiopia; ^19^ Department of Adult Health School of Nursing College of Health Sciences University of Health and Allied Sciences Volta Region Ghana; ^20^ Department of Social and Behavioural Science School of Public Health University of Ghana Accra Ghana; ^21^ College of Medicine and Allied Health Sciences Freetown Sierra Leone; ^22^ College of Public Health University of the Philippines Manila Philippines; ^23^ West Visayas State University Iloilo City Philippines; ^24^ Department of Medical Education Faculty of Medicine University of Colombo Colombo Sri Lanka; ^25^ Global Health Institute Wuhan University Wuhan China; ^26^ Office of the University President Catanduanes State University Virac Catanduanes Philippines; ^27^ University of Puthisastra Phnom Penh Cambodia

**Keywords:** global health, health challenges, health priorities, public health

## Abstract

The year 2022 saw COVID‐19 as the primary public health concern, with vaccine rollout and mandates at the forefront. Other viral infectious diseases, such as Monkeypox and Ebola, emerged as public health concerns. Climate change and political conflicts significantly impacted global health, increasing the demand for humanitarian assistance and protection. In 2023, it remains crucial to identify global and public health priority areas to coordinate and implement effective solutions. Through discussions with public health practitioners and researchers, we have identified key priority areas for 2023, namely, health systems, the mental health crisis, substance abuse, infectious diseases, malnutrition and food insecurity, sexual and reproductive health challenges, environmental pollution, the climate crisis, cancer, and diabetes. These priority areas highlight shared concerns that should be addressed to facilitate proactive and innovative health interventions and practices. To achieve universal healthcare targets for 2030, prioritization, financial investment, international cooperation, and collaboration in addressing these global health challenges are crucial. This requires coordination among public health decision‐makers, the private health sector, and opinion leaders to implement country‐specific healthcare financing and food security measures. Research, scientific knowledge, and technical capacities must be leveraged to produce sustainable interventions that effectively reduce health disparities and improve health system responsiveness to prevent these challenges from progressing to public health emergencies.

## INTRODUCTION

As the New Year begins, response measures to public health challenges are crucial for global human development. COVID‐19 was the main focus in 2022, with the rollout of vaccines and vaccination mandates at the forefront. The potential for herd immunity in the light of emerging virus variants and the evaluation of the transmission‐stopping capabilities of available vaccines was a major concern. Other viral infectious diseases such as Ebola and Monkeypox also represented significant global public health concerns [1]. Political conflicts, such as the Russia–Ukraine conflict, had global impacts, leading to soaring food and energy prices, migrant and refugee crises, inordinate health systems effects, and a potential nuclear disaster [2].

The 2022 list of global public health challenges [3] included COVID‐19, human resources for health, health financing, conflict and humanitarian crises, mental health, poverty, climate change, child health, reproductive health, and the global infodemic. Responses to COVID‐19 and emerging infectious diseases drove the concept of collaborative intelligence, optimizing disease surveillance and developing capacity for locally manufacturing vaccines. As projected, the consequences of the climate crisis were notable in 2022, with countries in Europe experiencing their hottest summers [3], deadly floods in Pakistan and Nigeria, and widespread droughts across Africa [4], Sexual and reproductive health rights were fiercely debated with the landmark ruling of the US Supreme Court to overturn *Roe v. Wade*. The 2022 list of global public health challenges also projected the already obvious high requirement for humanitarian assistance and protection due to political conflicts and natural disasters.

The world will continue to experience an influx of global health challenges in 2023. To proactively plan for and address these complex issues, it is crucial to identify global and public health priority areas. This allows the global community to coordinate, implement, and scale up local and international collaborative actions. Identifying these important challenges is integral to monitoring and developing policies to address these health risks, focusing on lessening the burden in low‐ and middle‐income countries (LMICs).

In developing this study, we engaged public health practitioners, experts, and researchers across sectors in discussions to identify and prioritize global public health priorities for 2023. Corroborating the responses with existing literature, we provide analysis and evidence to support public health priority areas for 2023. These highlight shared concerns facing human health and development that should be addressed to facilitate proactive, collaborative, and innovative health interventions and practices.

### Health systems

Healthcare systems are crucial in implementing health actions that promote population health and Sustainable Development Goals (SDGs). However, achieving universal health coverage (UHC) is impossible without the proper actions to strengthen health system challenges, especially in developing countries. The COVID‐19 pandemic has exposed the weaknesses in many health systems across high‐, middle‐, and low‐income countries. It has led to the overburden of healthcare systems and human resources for health, limiting the capacity of the systems to deliver essential services to communities [5]. Inadequate access to healthcare systems is a threat to global health: At least 50% of the world's population does not have access to the essential healthcare services they need [5]. In addition, due to uninsured medical costs, nearly 100 million individuals have been forced into extreme poverty annually due to the burden of out‐of‐pocket expenditures [6].

Health systems challenges vary across the globe, with developing countries experiencing more problems in health financing, health workforce development, infrastructures, supplies, healthcare information systems, and other vital aspects [7]. Developed countries also experience significant gaps in health systems concerning prescription drugs, the high costs of long‐term care, mental health services, dental services, and eye care [8]. Chronic global shortages of health workers are also prevalent due to an uneven supply of healthcare workers across countries further compounded by a significant migration of workers from developing to developed countries [9]. Health workforce shortages contribute to health inequities, which affect vulnerable populations, including women and children, rural communities, and marginalized groups [10]. This shortage affects the ability of healthcare systems to deliver essential services, including maternal and child health, infectious disease control, and noncommunicable disease management [11].

At a time when countries have yet to recover from the impacts of COVID‐19, a surge of emerging infectious diseases poses significant public health challenges that need to be adequately addressed [12]. Strengthening Primary Health Care (PHC) systems becomes vital to address health issues effectively and holistically, especially in regions where physical access through road and transport is a major problem [6]. PHC is essential for improving a health system's ability to withstand crises by empowering the local community in safeguarding public health, assisting in the surveillance of outbreaks and epidemics, and rapid responses to spikes in service demand. PHC is the “front door” of the healthcare system; an efficient PHC system lays the groundwork for improving crucial public health functions to address public health emergencies and early identification of people requiring clinical care [6].

### Mental health

One of the lasting impacts of the COVID‐19 pandemic is the decline in mental health. Historically, mental health and disorders were not a global health priority, focusing more on communicable and noncommunicable diseases [13]. In many countries, the management of mental health disorders has been isolated from conventional healthcare, with little funding relative to the disease burden [14]. Mental health concerns have risen post‐pandemic, with an incidence level higher than cancer [15]. There were disruptions in the delivery of mental healthcare and suicide prevention services during the pandemic. The 2022 WHO World Mental Health report indicated that the COVID‐19 pandemic triggered a 25% increase in the global prevalence of anxiety and depression [13]. The combinations of factors such as the rising cost of living, ongoing global conflicts, and the impact of climate change are driving the epidemic of anxiety and depression worldwide. The Russia–Ukraine conflict is estimated to have put nearly 10 million people at risk of mental health disorders like acute stress, anxiety, depression, and post‐traumatic stress disorder [16].

The global community must act toward the inclusion and funding of mental health support programs that extend beyond the pandemic. Despite countries only allocating less than 2% of national health budgets to mental health, funding has been received from both domestic and international contributions [17]. These must then be used to promote new models of interventions that integrate mental health support throughout the healthcare continuum. This transformation will strategically position mental healthcare and provide more effective support. The models that can be used include collaborative care models, digital health interventions, and peer support programs.

### Substance abuse

Today, alcohol and tobacco are legal and easily accessible to adults in most countries. However, substance use, excluding alcohol and tobacco, is responsible for approximately 500,000 deaths annually, with tobacco and alcohol estimated to result in over 8 million deaths annually [14, 18, 19]. Recreational drug use was also estimated to be responsible for over 42 million years of healthy life loss (disability‐adjusted life years, DALYs) in 2017, making up approximately 1.3% of the global burden of disease (GBD) [18]. It is estimated that about 11 million people globally inject drugs regularly. This includes 1.4 million infected with HIV and 5.6 million with hepatitis C [18, 19].

Substance abuse and its associated harms have contributed significantly to the shift from infectious diseases to noninfectious diseases in the GBD [14]. The GBD study estimated that in 2017, alcohol and drug use accounted for 5.1% and 0.9% of the GBD, respectively [20]. According to the 2021 World Drug Report by the United Nations Office on Drugs and Crime (UNODC), around 275 million people globally used drugs at least once in 2020, an increase of 22% over the last decade [21].

To address this serious public health challenge, a more proactive and innovative approach is needed, as the traditional ways of criminalization and punishment have not been effective. Stakeholders need to scale up funding and evidence‐based harm reduction interventions while increasing access to treatment and support for those struggling with substance abuse. Community education programs, recovery support services, and exercise and nutritional‐based interventions expand interventions for addressing substance abuse. Additionally, a more holistic approach is needed, targeting the underlying social and economic factors that contribute to substance abuse, such as stigma, poverty, unemployment, violence, and conflict. By aligning drug and substance abuse interventions with the general scope of mental health disorders, we can develop a system of holistic health protection.

### Infectious diseases

Emerging and reemerging infectious diseases present significant obstacles to improving health in poor communities and have a significant global impact. Pandemics can occur unexpectedly and spread rapidly, with the COVID‐19 pandemic serving as a reminder of the importance of preparedness and rapid response to emerging microbial threats [22]. Socioeconomic determinants and inequity can influence the occurrence and spread of infectious diseases, affecting access to healthcare, sanitation, and nutrition. Poverty and limited access to clean water and sanitation can contribute to the spread of infectious diseases such as cholera and diarrheal diseases [22]. Furthermore, ongoing issues such as HIV/AIDS and malaria have put significant pressure on healthcare systems.

Although significant progress has been made with regard to HIV/AIDS treatment, much remains to be done to ensure that all those living with HIV receive the care and treatment they need [23]. High‐burden countries such as Africa face unique challenges in responding to HIV, including the risk of transmission through injection drug use, lack of accessibility to antiretroviral therapy, and continued social stigma associated with drug use [24]. Similarly, malaria disproportionately affects the poorest and rural populations in Sub‐Saharan Africa, with disruptions in healthcare supply and demand further exacerbating the burden of the disease [25].

The expanding scope of infectious diseases calls for improving the quality of primary health services in healthcare delivery. Resilient and functional primary healthcare sets the foundation for boosting health promotion and services focusing on preventing infectious diseases. Providing adequate financial support to the public health infrastructure and working toward alleviating poverty are all crucial components of an effective response to emerging and reemerging infectious disease threats. Marked inequalities within and between countries slow down progress in the responses to infectious diseases, necessitating interagency and international collaborations for research, optimization of surveillance and response systems, and the shared goal of improving access to quality and affordable health services.

### Malnutrition and food safety

Malnutrition affects 2.36 billion adults [26], corresponding to approximately 29% of the global population. Malnutrition is a major issue among children: It is estimated that as of 2020, stunting (too short for age) incidents reached 149 million children under 5 (21%), whereas wasting (too thin for height) incidents reached 45 million (6%) [26]. Forty‐five percent of mortality under the age of 5 is linked to undernutrition, mostly occurring in LMICs [26]. Conversely, overnutrition has also become a threat to public health. Globally, approximately 39% of adults aged 18 years and over were overweight, and 13% were obese [26]. Malnutrition is linked to several socioeconomic factors, such as limited access to healthy food and a lack of awareness of what constitutes a healthy diet. Moreover, a healthy diet is becoming increasingly unaffordable, with food prices increasing by 11% between 2020 and 2021 globally [27]. The COVID‐19 pandemic has further increased the incidence of wasting to 60 million in 2022 due to reduced access to food [28]. The risk of undernutrition is further heightened due to ongoing conflicts, such as the Russia–Ukraine conflict, which has led to a 20% increase in food prices globally [27, 28]. Moreover, climate‐driven disasters have, to some extent, disrupted the food supply chain. All these factors have contributed to a more prominent risk of malnutrition in 2023.

Food safety is also a significant public health challenge with almost 1 in 10 people becoming ill after consuming contaminated food every year. This disproportionately affects LMICs and results in a global annual burden of 33 million DALYs and 420,000 premature deaths. The globalization of the production and supply of food has caused a wider and faster transmission of foodborne pathogens, including those that are resistant to antimicrobials [29]. Governments around the world have taken a number of measures to improve food security and safety. In 2022, the United Nations (UN) established the Global Crisis Response Group on Food, Energy, and Finance to address crises related to the Russia–Ukraine conflict [30]. Funding has been allocated to assist countries that are most at risk of famine [31]. In October 2022, the WHO published the Global Strategy for Food Safety 2022–2030, the third published strategy since 2002 [29]. The strategy emphasizes a One Health approach, involving collaboration and communication among stakeholders in food production, processing, and distribution to identify and mitigate safety risks, as well as implement improved surveillance, monitoring, and risk assessment systems to prevent, detect, and respond to foodborne illnesses. Although the impacts of these efforts remain to be seen in 2023, this holistic approach can help address food safety challenges more effectively by recognizing the interconnectedness of human, animal, and environmental health.

### Sexual and reproductive health

The unmet needs of sexual and reproductive health can lead to the illness and death of millions [32]. Some of the major Sexual and Reproductive Health and Rights (SRHR) challenges include inadequate comprehensive sexuality education (CSE) [33], gender‐based violence [34], teenage pregnancy [3], early marriage [35], sexually transmitted infections (STIs) [36], maternal health [37], unsafe abortion [38], unmet needs of family planning [39], infertility and reproductive cancers [40], and poor access to SRHR services [3]. Integrating CSE into the national curricula and educational systems is a significant challenge for many countries, especially for LMICs [33]. This deficiency is a major driver of adolescent pregnancies, estimated to occur annually in 21 million girls aged 15–19 in developing countries, as approximately 12 million of them give birth annually [3, 33]. In addition, more than 12 million girls each year get married before reaching the age of 18 [35]. Although global trends in child marriage have decreased, no region is on track to meet the SDG 5.3 target of eliminating this practice by 2030 [35]. Approximately 800 women die daily from pregnancy and childbirth‐related causes, around 45% of all abortions are unsafe, and more than 270 million women still have an unmet need for family planning [37, 38].

Challenges such as gender‐based violence and STIs have also increased in recent years. Gender‐based violence is a significant challenge that affects one in three women in their lifetime [34]. Gender‐based violence increased during the COVID‐19 pandemic, revealing a shadow pandemic of sexual gender‐based violence and menstrual health inequities [41]. For STIs, the 2019 GBD study depicted an increase in prevalence by 58.15% by 2019 from 1990 [42]. Each day, more than 1 million people become infected from four curable STIs: Syphilis, Gonorrhea, Chlamydia, and Trichomoniasis [36]. By the end of 2021, an estimated 38.4 people were living with HIV, and two thirds were from Africa [43]. With the COVID‐19 pandemic, the quality and access to services and treatment for these diseases have only worsened [3].

Achieving UHC through improved access to equitable, affordable, and nondiscriminatory SRHR information and services is key to improving demographic outcomes and fostering sustainable development. Multistakeholder engagements are needed to address these challenges in 2023, particularly through integrating SRHR information and services into healthcare delivery, educational systems, gender development, climate action, sociopolitical/community engagement, laws, policy design, and implementation.

Investment in SRHR is critical to surmounting this challenge in 2023. Communities and governments, especially those in LMICs, should improve domestic funding for SRHR to increase the present US$6.3 billion annual resources in 2020 to the required US$10.8 billion by 2030. A transition from the current donor‐dependent funding landscape to self‐financing systems is necessary to sustain the progress made in SRHR and global health.

### Environmental pollution

Globally, there is an increase in the risk exposure for attributable mortality and years of life lost to disability related to environmental pollution, particularly for ambient particulate matter pollution [42]. The WHO estimates that 7 million deaths are attributable to the joint effects of environmental and household air pollution. It is estimated that nearly the entire global population breathes air that exceeds the WHO‐recommended guidelines on air particulate matters, with LMICs suffering from the highest exposures [44].

The pollution of the environment is a major threat to humans, wildlife, and their habitats on land, in waterways, and in the oceans. The ingestion of microplastics and plasticizers in humans may be associated with infertility, obesity, endocrine dysfunctions, and malignancy [44]. The health impacts of using toxic heavy metals such as mercury and lead in fuel and paint products continue to be a significant problem in countries that have yet to adopt appropriate alternatives. This is just one aspect of the larger issue of anthropogenic pollution, or pollution stemming from human activity [44]. Human activities continue to pollute soil, water, and air, often without adequate monitoring and regulation, particularly in developing countries. The pollution of the ecosphere harms the health of humans and other living organisms and may also trigger political and security threats. It is crucial that we take immediate action to address these issues and implement sustainable solutions to mitigate the effects of anthropogenic pollution.

### Cancer

Cancer is a leading cause of death worldwide, accounting for 19.3 million new cancer cases and almost 10.0 million cancer deaths in 2020 [45]. The burden of cancer incidence and mortality is influenced by various risk factors, including lifestyle habits, environmental factors, and genetics [46]. The lack of access to timely access to cancer screening, detection, and treatment in many countries contributes to the high mortality rate associated with cancer [45]. Infections also play a significant role in cancer incidence, and cancers related to infections are potentially preventable. *Helicobacter pylori*, human papillomavirus, and hepatitis B and C viruses are common infectious agents linked to cancer. However, access to early identification and treatment of these infections is challenging in many developing countries.

Preventing cancer mortality is one of the SDGs for reducing premature mortality caused by noncommunicable diseases. The factors contributing to cancer‐related DALYs are age, tobacco, alcohol, dietary risks, environmental pollution, obesity, and occupational risks [47]. Screening and early detection require increased awareness of the first signs of cancers among the general public and improved accessibility and access to diagnostic services. Correct and timely diagnosis of cancers followed by effective treatment can preserve the quality of life of many. However, the costs of cancer therapies remain prohibitively high in many countries. For those with terminal stages of cancer, there is a strong need for palliative care treatments to relieve the symptoms and suffering of patients and their families.

### Climate change

The World Health Organization identifies climate change as the most significant health threat facing humanity in the intermediate term [48]. With increasing temperatures and other climate change‐related environmental issues, the global disease profile is changing. First, more infectious diseases are emerging and/or reemerging in certain regions, such as dengue in North and South America and Ebola in Africa. The climate is becoming increasingly suitable for the growth of disease vectors such as *Aedes aegypti*, with transmission increasing by almost 10% between 1950 and 2018 [49]. Additionally, climate change also results in more frequent and severe environmental disasters. The 2022 monsoon flooding in Pakistan affected over one third of the country's population and was thought to be particularly severe due to climate change [50]. Heat waves are also becoming more common, with a UNICEF report estimating that 23% of children (538 million) are exposed to high heat wave frequency, and by 2050, 1.6–1.9 billion children will be affected, threatening their health and well‐being [51]. Heat waves are associated with greater risks of respiratory and cardiovascular diseases, reduced access to quality food and nutrition, and a higher risk of mortality [51]. A study found that the odds of heat stroke hospitalization increase by 37% for every additional day of heat wave exposure, highlighting the serious impact of heat waves on the health outcomes of older adults [52].

Despite these impacts of climate change, the inclusion of health into climate management programs (and vice versa) is still minimal. Health has only been included as a global goal on climate adaptation at the 27th UN Climate Change Conference of Parties (COP27) in 2022 [53]. The real impact of this decision on the climate health landscape remains to be seen. In addition, although the WHO includes health‐specific climate financing as one of the 10 key components of climate‐resilient health systems [54], a separate funding facility for health within the climate financing framework has yet to be pledged. Because of this, climate change remains one of the top public health challenges that must be dealt with utmost urgency.

### Diabetes

Diabetes is a serious and potentially debilitating condition with costly complications to individuals, families, healthcare systems, and national economies. Globally, an estimated 536 million people are living with diabetes, with 783 million individuals projected to have the disease by 2045 [55]. According to WHO, approximately 95% of all adults with diabetes have Type‐2 diabetes, with an expected increase in percentage in coming years [56]. Although Type‐1 diabetes is a significant health concern, the GBD caused by Type‐2 is much greater due to its higher prevalence, association with preventable risk factors, and numerous complications. About 80% of adults with diabetes live in LIMCs [57]. Despite diabetes long being associated with overweight and obese individuals, the increasing prevalence among non‐overweight patients, particularly those of Asian and African ancestry, presents a peculiar challenge [58].

Prioritizing diabetes in 2023 is vital, as the present scope of food insecurity, economic downturn, and health systems changes increase the risk of this disease. A global shift in health systems paradigms for prevention and care efforts is essential to stem the tide of Type‐2 diabetes mellitus (T2DM) and its complications. Increased investment in patient self‐management education and support is integral to preventing and reducing the risk of complications and the impact of chronic disease. Effective T2DM management requires addressing contributing socioeconomic factors, promoting global equity, and expanding access to diabetes care in LMICs. These measures will ensure access to necessary care for T2DM, enable equitable distribution of healthcare resources and lead to better health outcomes.

## CONCLUSION

Identifying the top public health challenges is key to developing responsive, effective, and sustainable health delivery systems. As the conundrum of global health challenges expands, a high degree of prioritization, financial investment, international cooperation, and collaboration in tackling these challenges is needed to accomplish 2030 universal healthcare targets. This calls for a clear agreement among public health decision‐makers, the private health sector, and opinion leaders to drive the implementation of country‐specific measures for healthcare. These top 10 challenges are expected to dominate globally in 2023 and must therefore take some precedence in global health efforts. Leveraging research, fostering the evolution of scientific knowledge, and increasing technical capacities must be done as early as possible to produce sustainable interventions that can effectively adapt to both current and emerging public health issues. With the COVID‐19 pandemic underscoring the importance of investing in public health infrastructure and systems, global efforts must be started now before these public health challenges become their own public health emergencies.

## AUTHOR CONTRIBUTIONS

*Data curation; project administration; supervision; writing—original draft; writing—review and editing*: Deborah Oluwaseun Shomuyiwa. *Data curation; project administration; writing—original draft; writing—review and editing*: Thinley Dorji. *Writing—original draft; writing—review and editing*: Goodness Ogeyi Odey, Adriana Viola Miranda, Isaac Olushola Ogunkola. *Data curation; formal analysis; writing—original draft; writing—review and editing*: Yusuff Adebayo Adebisi. *Writing—original draft; Writing—review and editing*: Junjie Huang. *Writing—review and editing*: Sarawut Thepanondh, M. D. Lamawansa, Samrawit Solomon, Lydia Aziato, Philip Baba Adongo, Indika Mahesh Karunathilake, Hao Li, Martin C. S. Wong, Joseph Christian Obnial, Patrick Alain Azanza.

## CONFLICT OF INTEREST STATEMENT

Don Eliseo Lucero‐Prisno III and M. B. N. Kouwenhoven are Editorial Board members of Public Health Challenges and are coauthors of this article. Deborah Oluwaseun Shomuyiwa, Thinley Dorji, Goodness Ogeyi Odey, Adriana Viola Miranda, Isaac Olushola Ogunkola, Yusuff Adebayo Adebisi, Junjie Huang, Lin Xu, and Joseph Christian Obnial are Youth Editorial Board members of Public Health Challenges and a coauthor of this article. To minimize bias, they have been excluded from all editorial decision‐making related to the acceptance of this article for publication.

## ETHICS STATEMENT

No ethics approval was required.

## Data Availability

No database or primary data was used in preparing the manuscript.
